# Safety and Stability of Pulmonary Function in Patients with Decreased Respiratory Function Treated for Spasticity with OnabotulinumtoxinA

**DOI:** 10.3390/toxins12100661

**Published:** 2020-10-19

**Authors:** Ziyad Ayyoub, Allison Brashear, Marta Banach, Robert Schoene, William Stringer, Terry Boodhoo, Irina Yushmanova, Rozalina Dimitrova, Mitchell F. Brin

**Affiliations:** 1 Rancho Los Amigos National Rehabilitation Center, Downey, CA 90242, USA; zayyoubmd@msn.com; 2 Clinical Professor of Medicine, David Geffen School of Medicine at University of California, Los Angeles, CA 90095, USA; 3 Department of Physical Medicine and Rehabilitation, Western University of Health Sciences, Pomona, CA 91766, USA; 4 Department of Neurology, University of California, Sacramento, CA 95817, USA; abrashear@ucdavis.edu; 5 Department of Neurology, Jagiellonian University, 31-007 Krakow, Poland; martabanach@yahoo.com; 6 Sound Physicians, San Francisco, CA 95116, USA; rbschoene@gmail.com; 7 Lundquist Institute for Biomedical Innovation at Harbor-UCLA Medical Center, Torrance, CA 90502, USA; Stringer@ucla.edu; 8 Allergan plc, an AbbVie Company, Irvine, CA 92612, USA; Boodhoo_Terry@allergan.com (T.B.); Yushmanova_Irina@allergan.com (I.Y.); Dimitrova_Rozalina@allergan.com (R.D.); 9 Department of Neurology, University of California, Irvine, CA 92697, USA

**Keywords:** onabotulinumtoxinA, botulinum toxin type A, pulmonary function testing, spasticity, respiratory function, forced vital capacity, forced expiratory volume

## Abstract

Two randomized, placebo-controlled studies evaluated the pulmonary function safety of onabotulinumtoxinA (onabotA) for treatment of upper and/or lower limb spasticity. Patients with stable baseline respiratory status received one or two treatments with placebo, 240 U, or 360 U of onabotA. Pulmonary function tests, adverse events, and efficacy were measured at least every 6 weeks for 18 weeks (Study 1) or 30 weeks (Study 2). Study 1 enrolled 109 patients (*n* = 36–37/group) and Study 2 enrolled 155 patients (*n* = 48–54/group). Mean baseline forced vital capacity (FVC) was 76–78% of predicted per group in Study 1 and 71% of predicted per group in Study 2. In Study 1, change from baseline FVC values were significantly (*p* < 0.05) decreased vs. placebo at weeks 3 (240 U −57 mL vs. placebo +110 mL) and 12 (360 U −6 mL vs. +167 mL placebo). In Study 2, change from baseline FVC values were significantly decreased in the 360 U group vs. placebo at weeks 6 (−78 mL vs. +49 mL placebo), 13 (−60 mL vs. +119 mL placebo), 18 (−128 mL vs. +80 mL placebo), and 24 (−82 mL vs. +149 mL placebo). Individual pulmonary function-related adverse events were not correlated with PFT decreases. The most frequent pulmonary-related adverse events were nasopharyngitis (Study 1) and upper respiratory tract infection (Study 2). Ashworth scores were significantly improved at multiple time points in both studies. Injection of onabotA for spasticity in patients with decreased pulmonary function, at single and repeated doses of up to 360 U, was associated with small but statistically significant decreases in FVC or forced expiratory volume 1 s (FEV1) (>12% and 200 mL) that were subclinical and not correlated with any adverse clinical pulmonary events.

## 1. Introduction

OnabotulinumtoxinA (onabotA) is a biological medication that is injected into overactive muscles for the treatment of spasticity. The active protein, botulinum toxin type A, binds to acceptor sites on cholinergic nerve terminals and is internalized into the cell where it inhibits acetylcholine release, thereby reducing muscle overactivity. Randomized, controlled trials have found that onabotA is superior to placebo for the treatment of adult spasticity [1,2,3] with an excellent safety profile. However, given the possible spread to distant muscles, concerns about the impact of botulinum toxin on pulmonary status remain.

OnabotA is administered locally and the clinical effects are expected to be limited to structures in the vicinity of the injection region. However, respiratory-related adverse events and respiratory dysfunction have been reported in some patients following injection into the limbs for the treatment of spasticity, although these are not always considered treatment related [4,5,6]. These changes may be due to systemic spread of toxin at high doses or from worsening neurologic status from spasticity, cognition, or other unrelated medical illness.

Patients may develop limb spasticity following damage to the descending motor pathways of the central nervous system caused by stroke, spinal cord or traumatic brain injury, and demyelinating diseases such as multiple sclerosis. These conditions can cause upper motor neuron syndrome, a constellation of signs that includes skeletal muscle weakness, altered muscle tone, and exaggerated deep tendon reflexes [7]. The neural damage can lead to medical complications in many different bodily systems, affecting cardiac, circulatory, urinary, and respiratory function. For instance, stroke patients often exhibit postural abnormalities that interfere with the coordinated action of peripheral and respiratory muscles [8]. Pneumonia and ventilatory dysfunction are common in stroke patients [9], who may also show alterations in cortico-respiratory pathways associated with degree of disability, site of infarction, and respiratory dysfunction [10]. Lung dysfunction, mucous retention, and pulmonary infections are also common in patients with cervical and upper thoracic spinal cord injuries [11]. These neurologic changes can also have an effect on pulmonary function testing performance.

Given the propensity in patients with spasticity to suffer respiratory complications, and the potential of onabotA to affect respiratory function, we performed a post hoc analysis of two studies that utilized this method to treat spasticity to determine the potential effect on pulmonary function test results, and assess if observed changes in pulmonary function testing affected the respiratory clinical outcomes (e.g., pneumonia and rhinosinusitis).

## 2. Results

### 2.1. Patient Demographics and Baseline Characteristics

**Figure 1:**
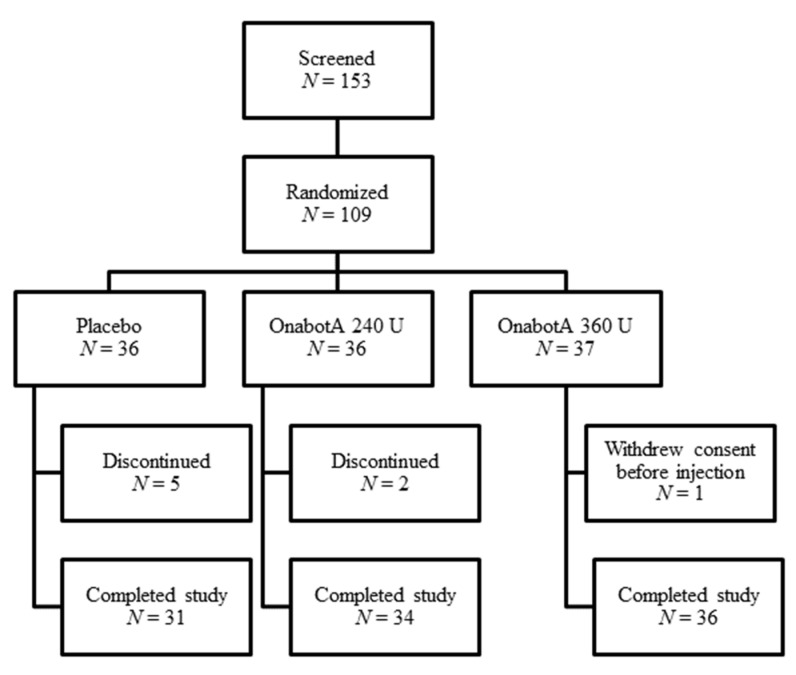
Study 1 patient disposition.

In Study 1, 153 patients were screened and 109 were randomized; patients were required to meet the inclusion/exclusion criteria (see Section 4) to be randomized into the study. A total of 101 patients completed the study (Figure 1).

**Figure 2:**
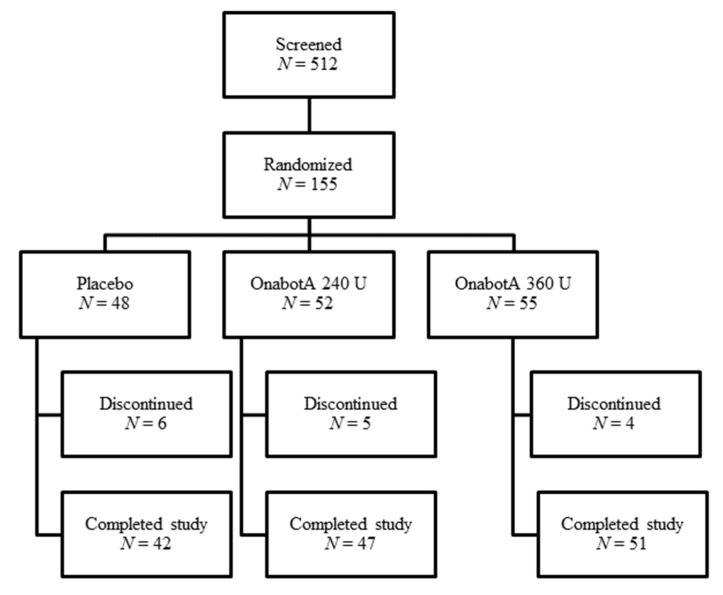
Study 2 patient disposition.

In Study 2, 512 patients were screened and 155 were randomized; patients were required to meet the inclusion/exclusion criteria (see Section 4) to be randomized into the study. A total of 140 patients completed the study (Figure 2).

Table 1 lists the demographic and baseline characteristics of patients included in the present analyses. Within each study, mean ages were similar for the onabotA and placebo groups. The majority of patients in the studies were Caucasian and the percentage of female patients ranged from 25% to 62% across treatment groups. The mean percent of predicted forced vital capacity (FVC) at study baseline ranged from 76% to 78% per group in Study 1 and was 71% for all groups in Study 2. The mean percent of predicted forced expiratory volume 1 s (FEV1) at study baseline ranged from 79% to 80% per group in Study 1 and from 65% to 66% per group in Study 2. In Study 1, all patients had post-stroke spasticity, per inclusion criteria. In Study 2, most patients had post-stroke spasticity (143/155; 92%), but spasticity was attributed to an etiology other than stroke in 3/48 (6.3%) of placebo patients, 6/52 (11.5%) onabotA 240 U patients, and 3/55 (5.5%) onabotA 360 U patients.

### 2.2. Muscles Injected

In Study 1, the most commonly injected muscles were flexor digitorum superficialis, flexor carpi radialis, flexor carpi ulnaris, flexor digitorum profundus, and biceps (Table A1).

A total of 85 patients were reinjected at week 12, and 18 were not reinjected. In Study 2, the most commonly injected muscles were biceps, brachioradialis, flexor carpi radialis, flexor carpi ulnaris, flexor digitorum profundus, and flexor digitorum superficialis (Table A2). A total of 110 patients were reinjected at week 12, 31 were reinjected at week 18, and 14 were not reinjected.

### 2.3. Pulmonary Function Tests

#### 2.3.1. Mean Change from Baseline FVC

In the double-blind placebo controlled (DBPC) Population of Study 1, mean decreases in FVC from study baseline were noted in the onabotA 360 U group at weeks 1, 12, 15, and 18 and in the 240 U group at weeks 1 and 3, and mean increases in the placebo group were noted at all time points (Table 2). Over the 18-week study, the ranges for mean change from study baseline for the 360 U, 240 U, and placebo groups were −25 to +47 mL, −57 to +58 mL, and +87 to +167 mL, respectively. In the 360 U group, a statistically significant difference between the treatment groups was observed at week 12 (−6 mL 360 U vs. +167 mL placebo) and, in the 240 U group, a statistically significant difference was noted at week 3 (−57 mL 240 U vs. +110 mL placebo). In the 360 U group, changes from baseline FVC ranged from −25 mL at week 15 to +47 mL at week 3; in the 240 U group, changes from baseline FVC ranged from −57 mL at week 3 to +58 mL at week 18; in the placebo group, changes from baseline FVC ranged from +87 mL at week 3 to +167 mL at week 12.

In Population 2 (reinjection at week 12) of Study 1, mean decreases in FVC from study baseline were noted in the onabotA 360 U group at weeks 1, 6, 12, 15, and 18 and mean increases in the placebo group at each time point; statistically significant differences between the 360 U and placebo groups were observed at week 6 (−11 mL vs. 177 mL) and week 12 (−11 mL vs. 170 mL) (Table A3). In the 240 U group, there were mean increases in FVC at most time points (+8 mL at week 18 to +37 mL at week 12) and none was statistically significant compared to placebo. In the 360 U group, changes from baseline FVC ranged from −21 mL at week 18 to +51 mL at week 3; in the 240 U group, changes from baseline FVC ranged from −5 mL at week 13 to +37 mL at week 12; in the placebo group, changes from baseline FVC ranged from +89 mL at week 18 to + 177 mL at week 6.

In the DBPC Population of Study 2, the onabotA 360 U group showed mean decreases in FVC from study baseline at each time point after week 1, whereas the placebo group showed mean increases at all time points after week 1 (Table 3). In the 360 U group, the maximum mean decrease from study baseline was −128 mL (−4%) at week 18 and, in the placebo group, the maximum increase was +149 mL (+5%) at week 24. Statistically significant differences between the 360 U and placebo groups were observed at weeks 6 (−78 mL vs. +49 mL), 13 (−60 mL vs. +119 mL), 18 (−128 mL vs. +80 mL), and 24 (−82 mL vs. +149 mL). The 240 U group also showed mean increases in FVC from study baseline at each time point, ranging from +52 mL (week 6) to +100 mL (week 12), and none was statistically significant compared to placebo. In the 360 U group, changes from baseline FVC ranged from −128 mL at week 18 to +20 mL at week 1; in the 240 U group, changes from baseline FVC ranged from +52 mL at week 6 to +100 mL at week 12; in the placebo group, changes from baseline FVC ranged from −1 mL at week 1 to +149 mL at week 24.

Results were similar in Population 2 (reinjection at 12 weeks) of Study 2 (Table A3). Statistically significant differences between the onabotA 360 U group and placebo groups were observed at weeks 13 (−60 mL vs. +119 mL), 18 (−132 mL vs. +112 mL), 24 (−106 mL vs. +186 mL), and 30 (−69 mL vs. +73 mL). In the 240 U group, there were mean increases in FVC at each time point, ranging from +31 mL (week 18) to +87 mL (week 1), and none was statistically significant compared to placebo. In the 360 U group, changes from baseline FVC ranged from −132 mL at week 18 to +35 mL at week 1; in the 240 U group, changes from baseline FVC ranged from +31 mL at week 18 to +87 mL at week 1; in the placebo group, changes from baseline FVC ranged from −6 mL at week 1 to +186 mL at week 24.

The overall pattern of results for the onabotA groups in Population 3 were similar to those of the DBPC Population and Population 2, with the 360 U group showing mean decreases from study baseline at all time points except weeks 24 and 30, and the 240 U group showing mean increases from study baseline at all time points (Table A3). However, the placebo group showed decreases from baseline at five of six time points. The only significant difference observed in either onabotA group compared with placebo in Population 3 was a significant increase in the 240 U group at week 12 (240 U: +163 mL vs. placebo: −275 mL). In the 360 U group, changes from baseline FVC ranged from −127 mL at week 18 to +36 mL at week 24; in the 240 U group, changes from baseline FVC ranged from +72 mL at week 6 to +189 at week 19; in the placebo group, changes from baseline FVC ranged from −275 mL at week 12 to +25 mL at week 19.

Change from baseline FEV1 data is presented in Appendix A Table A4 for Study 1 and Table A5 for Study 2. Results were generally similar to those with FVC, except that there were fewer statistically significant time points overall, and scores in the 240 U onabotA group tended to decrease in Study 1 instead of increase.

#### 2.3.2. Distribution of Change from Baseline FVC

**Figure 3:**
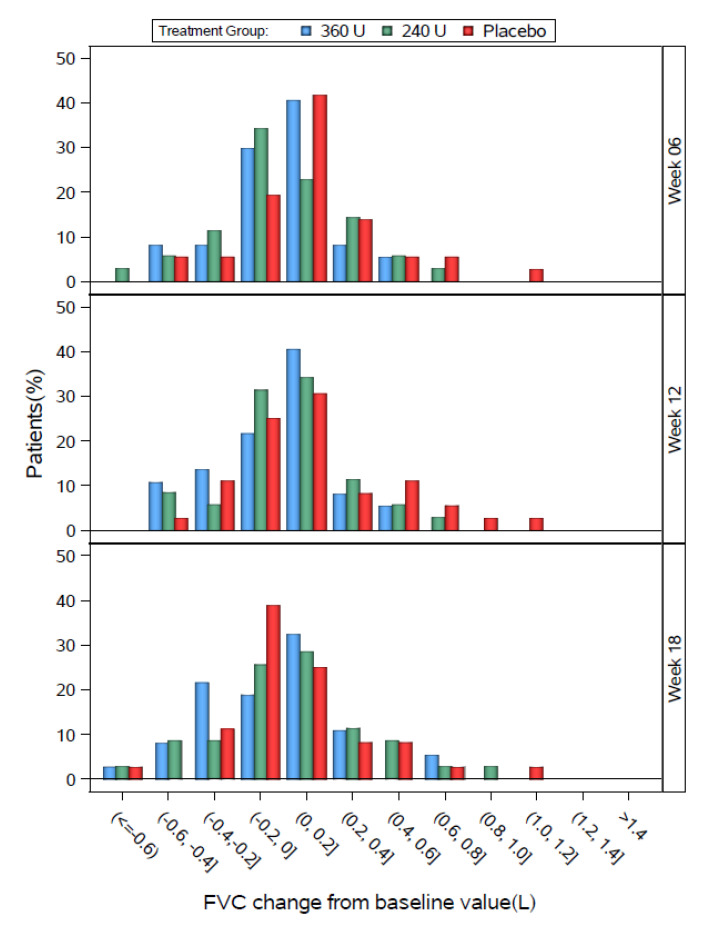
Study 1 FVC change from baseline by study visit for the DBPC Population.

**Figure 4:**
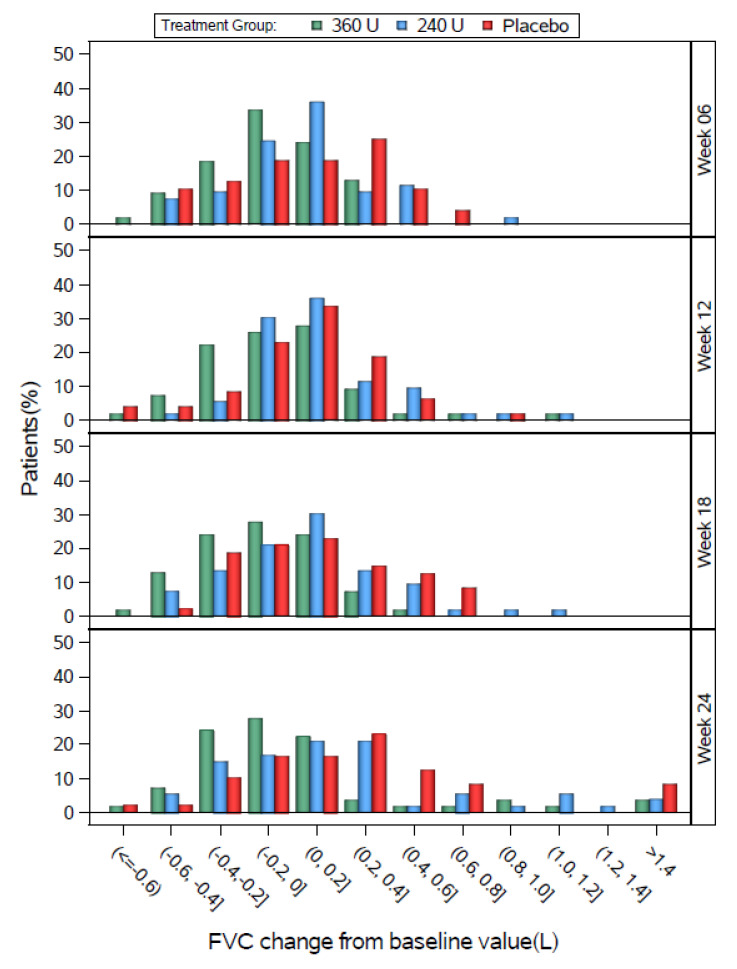
Study 2 FVC change from baseline by study visit for the DBPC Population.

The distributions of individual change from baseline FVC values for the DBPC populations are shown by 0.2 L increments in Figure 3 (Study 1) and Figure 4 (Study 2). In all groups in both studies, most patients showed changes from baseline FVC values between −0.2 and 0.2 L. In Study 1, the three groups had generally similar distributions, except that the 240 U group tended to shift slightly left at week 6, the placebo group tended to shift slightly left at week 18, the placebo group tended to have a greater % of patients at 0.4 L or higher at weeks 6 and 12, and the 360 U group tended to have a higher % of patients between −0.4 and −0.2 L at week 18.

In Study 2, the three groups had generally similar distributions, except that the 360 U group tended to shift slightly left relative to placebo at most weeks, and the peak percentage of the 240 U group was between 0 and 0.2 L at week 6, compared with a peak between 0.2 and 0.4 L for placebo at week 6.

#### 2.3.3. Percentage of Patients with Decrease of ≥12% and ≥200 mL in FVC or FEV1

In the overall DBPC Population of Study 1, the percentages of patients with decrease of ≥12% and ≥200 mL in FVC or FEV1 in the 360 U and placebo groups were relatively similar and stable over time (Table 4). The percentage of patients in the 240 U group meeting these criteria was also relatively stable but was higher than the other two groups at most time points. The pattern was similar in Population 2. No statistically significant differences were observed between the onabotA and placebo groups at any time point in any of the populations.

In the overall DBPC Population of Study 2, the percentages of patients with decrease of ≥12% and ≥200 mL in FVC or FEV1 in the 360 U group tended to be higher at weeks 12 through 30 than at earlier time points, whereas the percentage in the placebo group tended to remain stable or decrease (Table 5). In the 240 U group, the percentages of patients meeting these criteria tended to be higher at weeks 18 through 30 than at earlier time points. The patterns observed in the two onabotA groups were similar to those of the DBPC Population, but the percentages in the placebo group tended to be lower. Statistically significant differences between the 360 U and placebo groups were observed in the overall DBPC Population at week 24, and in Population 2 at weeks 12, 18, and 24. In Population 3, the placebo group tended to have the highest percentages of patients meeting these criteria at most time points, although no statistically significant differences were observed between the onabotA and placebo groups.

#### 2.3.4. Change from Baseline FVC Stratified by Baseline FVC

**Figure 5:**
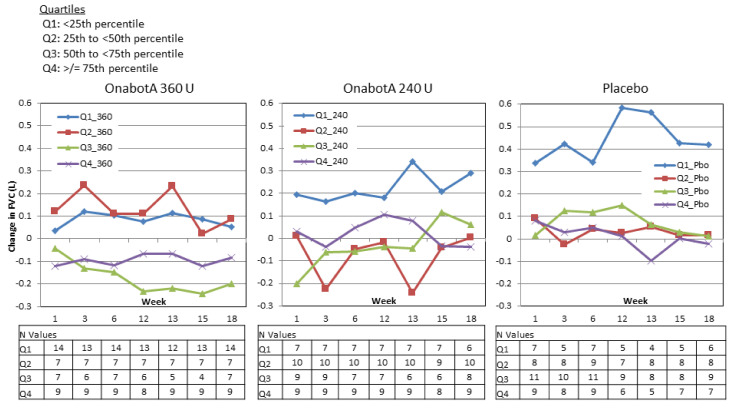
Study 1 change from baseline FVC by baseline FVC quartile.

In Study 1, no predictable pattern of FVC increases or decreases were discernible based on FVC quartile at baseline (Figure 5). In the 360 U group, FVC tended to slightly increase in patients with the lowest baseline FVC values and tended to slightly decrease in patients with the highest baseline FVC values. In the 240 U and placebo groups, FVC tended to increase in patients with the lowest baseline FVC and tended to show smaller, variable increases and decreases in the other groups.

**Figure 6:**
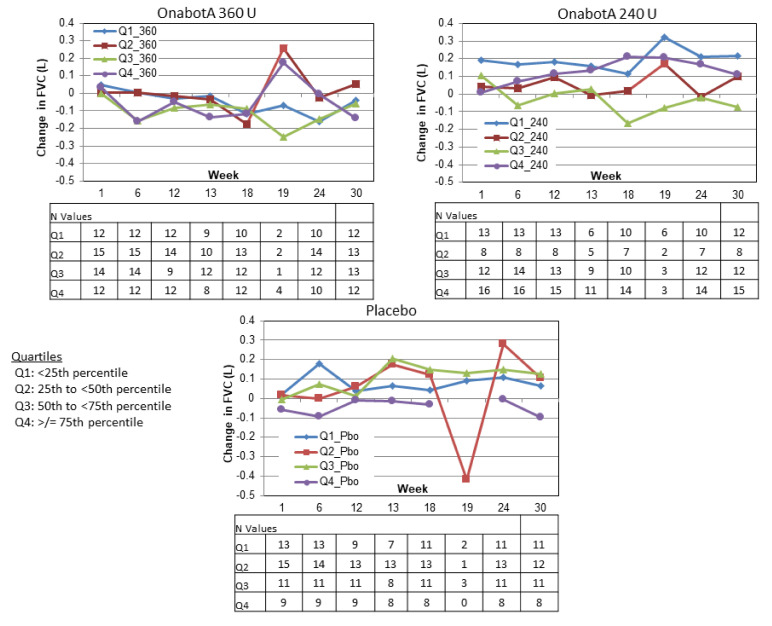
Study 2 change from baseline FVC by baseline FVC quartile.

Similarly, in Study 2, no predictable pattern of FVC increases or decreases were discernible based on FVC quartile at baseline (Figure 6). In the 360 U group, FVC tended to slightly decrease regardless of baseline FVC values (note that week 19 is an exception and includes only the few patients who were reinjected at week 18; see *N* values in Figure 6). In the 240 U and placebo groups, FVC tended to slightly increase regardless of baseline FVC. Again, week 19 tended to be an exception and included very small numbers of patients.

### 2.4. Efficacy

In Study 1, Ashworth scores were significantly reduced in the upper and/or lower limbs of both treatment groups compared with placebo at weeks 1, 3, 6, 13, and 18, and in the 240 U group at week 15 (Table 6). Scores tended to be reduced at week 12 in both treatment groups and at week 15 in the 360 U group compared with placebo (*p* ≤ 0.064).

In Study 2, Ashworth scores were significantly reduced in the upper limbs of both treatment groups compared with placebo at weeks 1, 6, and 18, and in the 240 U group at week 12 (Table 7). Scores tended to be reduced at week 12 in the 360 U group compared with placebo (*p* = 0.079). Change in Ashworth scores for the separate joints are presented in the Appendix A (Table A6).

### 2.5. Safety

#### 2.5.1. Adverse Events

Overall, the rates of any adverse events, treatment-related adverse events, and serious adverse events were comparable across treatment groups within each study (Table 8). In Study 1, seven patients experienced serious adverse events (Table A7), and one patient discontinued due to an adverse event (placebo). In Study 2, 22 patients experienced serious adverse events (Table A8).

One patient in the 360 U group died of a cardiac arrest on day 31. Prior to the event, the patient had a 2–3 week history of increased dyspnea and persistent heartburn for which he had not sought medical attention. In the opinion of the clinical investigator, this adverse event was not related to the study medication. In addition to this patient, three others discontinued due to adverse events, one of which was considered related to treatment (exacerbation of preexisting chronic obstructive pulmonary disease in the 240 U group). There were no cases of distant spread of toxin.

#### 2.5.2. Pulmonary Related Adverse Events

Pulmonary related adverse events tended to be higher in the onabotA 360 U group than placebo in both studies, due mainly to nasopharyngitis in Study 1 and upper respiratory tract infection in Study 2; pulmonary-related adverse events that occurred in at least 3% of patients in any group are shown in Table 9 and Table 10, whereas less frequent events are shown in Table A9 for Study 1 and Table A10 for Study 2.

#### 2.5.3. Medical Review of Relationship Between PFT and Pulmonary Related Adverse Events

In Study 1, similar percentages of patients in the 240 U and placebo groups patients experienced ≥12% decrease and ≥200 mL decrease from baseline in either FVC or FEV1 at any time, whereas the percentage in the 360 U group was slightly lower: 360 U: *n* = 13/37; 35.1%, 240 U: *n* = 16/35; 45.7%, placebo: *n* = 15/36; 41.7%. Decreases of ≥12% and ≥200 mL from baseline in either FVC or FEV1 were not correlated with pulmonary related adverse events.

In Study 2, the highest percentage of patients with ≥12% decrease and ≥200 mL decrease from baseline in either FVC or FEV1 at any time occurred in the 360 U group, whereas percentages in the other groups were similar: 360 U: *n* = 28/54; 51.9%; 240 U: *n* = 21/53; 39.6%, placebo: 18/48; 37.5%. None of the 21 patients in the onabotA 240 U group and only one of the 28 patients in the 360 U group had a co-incidental pulmonary related adverse event (Bronchitis). This patient showed ≥12% decrease and ≥200 mL decrease from baseline in either FVC or FEV1 at three study visits. The patient reported that his Bronchitis began 120 days after the second injection. The event was only reported at the week 24 visit. Based on this timing, the event was medically evaluated as inconsistent with the pharmacology of botulinum toxin.

## 3. Discussion

These two PFT studies showed a pharmacodynamic effect of onabotA on pulmonary function tests (FVC and FEV1), but the present analysis did not uncover any new pulmonary function adverse events that were deemed related to onabotA. Further assessment of the pulmonary related adverse events at the individual patient level, as opposed to the study population level, did not demonstrate a correlation with PFT decreases. Overall, based on the available data set, the pharmacodynamic decreases observed were not clinically relevant.

Statistically significant differences between onabotA 360 U and placebo in FVC were observed at several time points, but the magnitude of the mean FVC decreases from baseline with onabotA 360 U were small (maximum decrease of −128 mL; −4.5%), and occurred in conjunction with small mean FVC increases from baseline with placebo (maximum increase of 189 mL; 8.2%) and onabotA 240 U (maximum increase of 167 mL; 8.3%). Differences between these groups were primarily observed in Study 2, and in the DBPC Population (all patients) and Population 2 (second injection at 12 weeks), but not the smaller Population 3 (second injection at 18 weeks). In both studies, changes in FVC with the 240 U dose of onabotA generally did not differ from placebo. Results with FEV1 were similar, with a maximum mean decrease from baseline with onabotA 360 U of −59 mL, −2.4%, and occurred in conjunction with small mean increases from baseline with placebo (maximum increase of 140 mL; 2.9%), and small mean decreases (Study 1; maximum decrease of 137 mL; −6.0%) or increases (Study 2; maximum increase of 214 mL; 9.8%) with onabotA 240 U.

Given the lack of specific guidelines for clinically important decreases in PFTs, we explored changes in the numbers of patients with ≥12% and ≥200 mL decrease from baseline in either FVC or FEV1. In Study 2, statistically significant differences between the 360 U and placebo groups were noted on this variable at several time points week 12 or later. These statistically significant differences were seen only in the population retreated at 12 weeks and not the population retreated at 18 weeks and were not evident at any time in Study 1.

These safety findings are consistent with the established safety profile of onabotA in the treatment of limb spasticity. For example, a pooled analysis of nine randomized, double-blind studies of onabotA for the treatment of upper and lower limb spasticity found that the majority of adverse events were mild or moderate in severity, with three adverse events reported by significantly more patients treated with placebo (injection site pain, chest pain, allergic reaction) and one adverse event reported by significantly more patients treated with onabotA (nausea) [5]. A systematic review and meta-analysis of 22 studies in which patients were treated with various botulinum toxin type A products for upper limb spasticity found no significant differences in the rate of adverse events compared with placebo [12]. A real-world study of 10 treatment centres in Germany that included 508 patients and 2005 treatment sessions of onabotA for spasticity found that minor to moderate side effects were reported in <1% [13]. Thus, when used at the studied doses, onabotA is well tolerated in this population of patients who often have multiple serious comorbidities.

An important related question is whether patients with greater respiratory compromise at baseline show larger decreases in PFTs following onabotA injection. Inspection of change from baseline FVC values by baseline FVC quartile for the 360 U group did not indicate such a relationship.

Several other studies have included PFTs evaluations following onabotA injections, although they were not the primary focus. One of these studies reported no clinically relevant changes in PFTs following onabotA for upper-limb spasticity (clinical relevance not defined) [14] and another reported a statistically significant decrease in FEV1 with 360 U of onabotA vs. placebo at one time point (18 weeks) [15]. A pooled analysis of two studies found no statistically significant difference between onabotA and placebo in the percentage of patients with at least 15% decrease from baseline FEV1 or FVC [5]. A study of up to 800 U incobotulinumtoxinA injected for limb spasticity reported that mean and median FEV1 values ranged from 82.5% to 85.1% at all time points [16]; units of onabotulinumtoxinA are non-interchangeable and are not equal to incobotulinumtoxinA units. These data are difficult to compare with the studies described here because they presumably refer to comparison with normative data (although not explicitly stated by the authors), as opposed to the change from absolute values used in the present studies. Additionally, patients in the Wissel study were required to have baseline FEV1 values of at least 70% of predicted, whereas patients in the present studies were required to have baseline FEV1 values of at least 50% of predicted (Study 1) or 40–80% of predicted (Study 2—i.e., this study was specifically designed to enroll patients with lower baseline FEV1 values). We felt that it was important to study a patient population with baseline pulmonary impairment because many patients who are treated with botulinum toxin type A will have pulmonary comorbidities. Another study examined botulinum toxin injection into the cricothyroid muscles for the treatment of bilateral vocal fold abductor paralysis [17]. In that study, the goal was to inhibit the cricothyroid muscle to permit repositioning of the vocal folds and ultimately improve ventilation. As predicted, the study found increases in FVC (from a mean of 1.9 to 2.4 L) and FEV1 (from a mean of 1.6 to 2.3 L) after onabotA treatment for patients who achieved unilateral vocal fold motion.

Although efficacy was not a primary outcome of the present studies, the Ashworth scale was used to measure muscle tone at each follow up time point. Consistent with other published studies [1,15], onabotA significantly reduced muscle tone at multiple postinjection time points.

Limitations of the present studies include the use of fixed doses, which did not allow physicians to adapt doses to each patient’s pattern of spasticity. The use of fixed doses is common in clinical studies, but in clinical practice, dose per muscle and injection session may vary based on each patient’s need and response to prior treatment. Another limitation of the present study was lack of agreement in the literature as to clinically relevant PFT decreases. Individual patient factors such as comorbidities are believed to influence clinical relevance [18], and thus there may not be a set PFT decrease that can be identified as clinically relevant for the mostly post-stroke population studied here. Consequently, the decrease in FVC or FEV1 of 12% and 200 mL was arbitrarily defined and is of uncertain relevance. Design related limitations include non-standardization of the two study protocols and injection sites (upper vs. lower limb, different muscles). Multiplicity of comparisons was not considered because these were primarily safety studies.

The relationship between central nervous system damage and respiration is complex. Stroke patients show impairments in the cortico-respiratory tract, posture, and balance, all of which can affect respiratory function [8,10,19,20]. Indeed, patients in the present studies had mean baseline FVC and FEV1 values ranging from 65% to 80% of predicted. By treating limb spasticity, onabotA may alter the dynamic between posture and breathing; FVC and FEV1 provide only a limited window into respiratory function and changes may be observed in some of the parameters not evaluated here (e.g., effort). Moreover, patients with central nervous system damage due to stroke, spinal cord injury, or multiple sclerosis show alterations in the immune system that predispose them to respiratory infection [21,22,23]. These findings suggest a complex interplay between pulmonary function, neurological symptomatology, and immune status in patients with central nervous system damage.

Overall, the present studies found that the pharmacodynamic decreases in FVC and FEV1 were not clinically relevant and not associated with pulmonary function-related adverse events in this primarily post-stroke patient population with decreased pulmonary function. These results are consistent with the established safety profile of onabotA in the treatment of limb spasticity, as documented in the literature.

## 4. Materials and Methods

### 4.1. Study Design and Procedure

Study 1 was conducted between March 2000 and February 2001 at 12 sites in the United States, and Study 2 was conducted between November 2003 and August 2009 at 34 sites in the United States and European Union. Both were phase 2, prospective, placebo-controlled studies in which patients were randomized to placebo, 240 U, or 360 U onabotA. Institutional Review Board or Independent Ethics Committee approval was obtained at each study site prior to study initiation (Nos. 191622-030 and 191622-057-03; Date: 13 May 2003).

Study 1 used a randomization block size of 6 and ratio of 1:1:2:2 (placebo 240 U, placebo 360 U, onabotA 240 U, and onabotA 360 U) to ensure that one placebo-treated patient (both doses) entered the trial for every onabotA 240 and 360 U patient (1:1:1). A central randomization process assigned a study number to each qualified patient based on FEV1 status (≥65% vs. <65% of predicted value) and the randomization scheme. An independent designee obtained randomization information for each patient and instructed the site as to which medication to inject. All vials were identical in appearance before and after reconstitution, and all study personnel were blind to treatment.

In Study 2, patients were randomized in a 1:1:1 ratio to receive placebo, onabotA 240 U, or onabotA 360 U. Randomization was stratified by baseline FEV1 into two cohorts: one with 40–60% predicted FEV1 and the other with 61–80% predicted FEV1. An independent designee assigned patient randomization numbers in chronological order. All vials were identical in appearance before and after reconstitution, and all study personnel were blind to treatment.

**Figure 7:**
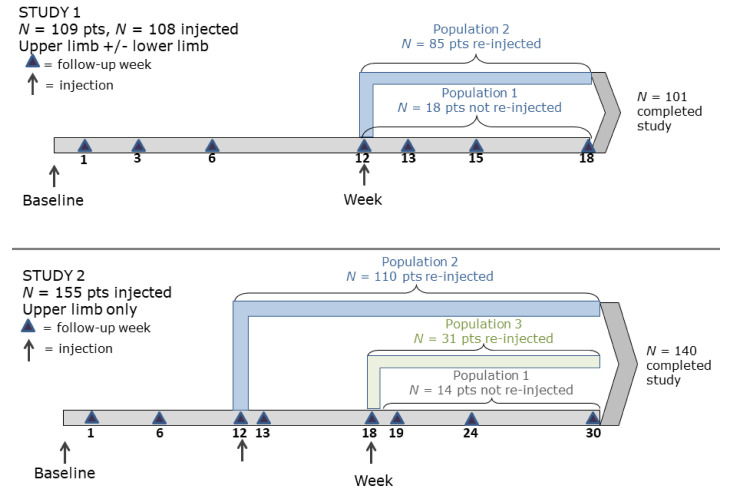
Study designs.

Patients received injections into the affected upper limb(s) (both studies) and/or lower limb(s) (Study 1 only) per the injecting physician’s discretion. Follow-up visits for Study 1 were weeks 1, 3, 6, 12, 15, and 18 (Figure 7). Patients with FEV1 ≥50% of predicted at week 12 (*n* = 85) were eligible to be reinjected with the same dose and into the same muscles as the first treatment. Patients reinjected at week 12 were also followed up at week 13. Eighteen patients did not receive a second injection: 10 because the investigator judged it not medically necessary, four because of adverse events related or unrelated to the study medication, three because of missed visits, and one unknown. Follow-up visits for Study 2 were weeks 1, 6, 12, 18, 24, and 30 (Figure 7). Patients may have been reinjected at weeks 12 or 18 if needed. Patients must have been reinjected with the same volume and dose and, preferably, into the same muscle(s) as the first treatment. Patients who were reinjected at week 12 were also followed-up at week 13, and patients reinjected at week 18 were also followed up at week 19. Fourteen patients did not receive a second injection: 11 because they discontinued before week 12 and three because the investigator did not feel that the patient had sufficient spasticity in the affected upper limb to warrant another treatment. Percent of predicted PFT values for both studies were based on those of healthy individuals [24].

The two studies were approved by the institutional review boards at each participating site and were conducted in accordance with the Declaration of Helsinki, the International Conference on Harmonization (ICH) Good Clinical Practice (GCP), and applicable regulatory requirements.

### 4.2. Outcomes

Pulmonary function tests (PFTs) were the primary outcome measures for both studies. Forced expiratory volume (FVC; amount of air that can be forcibly exhaled after taking the deepest breath possible) and forced expiratory volume in 1 s (FEV1) were prespecified as primary outcome variables for Study 2, and maximal inspiratory pressure (PI max) was defined as the primary outcome variable for Study 1. No statistically significant between-group differences were seen in PI max at any follow-up visit, and published evidence has documented high variability in PI max [25,26]. Thus, the present analysis focuses on FVC and FEV1 for both studies.

No accepted definition of a clinically important or clinically meaningful decrease in PFTs is available in the literature. The American Thoracic Society/European Respiratory Society (ATS/ERS) suggests that weekly changes in FVC of at least 11% in normal individuals and 20% in individuals with chronic obstructive pulmonary disease (COPD) are important, noting that significant statistical or biological changes vary by parameter, time period, and patient [18]. Current ATS/ERS guidelines state that a bronchodilator response of 200 mL plus a change (improvement) of 12% in either FEV1 or FVC is clinically important [18]. Therefore, given the lack of specific guidelines for clinically important decline in PFTs related to spasticity treatment, we explored a decrease of ≥12% and ≥200 mL from baseline in either FVC or FEV1 as the clinically meaningful change (decline) for this manuscript.

Efficacy was assessed in both studies using the 5-point Ashworth scale (0 = none to 4 = very severe). Adverse events (AEs) were monitored at each visit, beginning with a general question to the patient and followed by directed questioning and examination, if needed. Pulmonary function-related adverse events were medically reviewed for patients who showed ≥12% decrease and a ≥200 mL decrease from baseline in either FVC or FEV1 at any time to determine whether they were accompanied by decreases in PFTs at the same visit or approximately the visit time frame (“co-incidental”). PFTs were performed at defined visits but adverse events may have occurred preceding the visit. Patients treated with any dose of onabotA during the double-blind phase of the studies were included. For evaluation of temporal relationships, it was assumed that the onset of a pulmonary function-related adverse event between days 1 and 14 could potentially be related to onabotA given the medication’s pharmacology, though a 30-day window was used in this analysis to broaden the assessment. The event was evaluated using medical history, as well as the listed comorbidities and concomitant medications. The evaluation of these data is limited by the fact that the PFT measurements occurred at protocol-defined, routine study visits that did not necessarily coincide with the time of onset or presence of pulmonary function-related AEs occurring between visits.

### 4.3. Sample Size Determination

For Study 1, a sample size of 29 patients per treatment group was estimated to provide 80% power to show that the mean percent change in PI max from baseline was not inferior to placebo for the mean percent change in PI max from baseline. For Study 2, a sample size of 50 patients per treatment group was estimated to provide 85% power to show non-inferiority compared to placebo for the FEV1/FVC ratio.

### 4.4. Patients

All patients provided written, informed consent. Both studies included medically stable adults with spasticity in the upper and/or lower-limb(s) due to stroke (Study 1) or upper motor neuron syndrome (Study 2). Patients in Study 1 must have been at least 6 weeks post stroke and stroke patients in Study 2 must have been at least 6 months post stroke. Patients must have been a candidate for a dose of 360 U onabotA in the affected limb based on the investigator’s judgment. Study 1 required that patients have baseline FEV1 ≥50% of predicted values and no history of acute pulmonary complications within the past 6 weeks. Study 2 required that patients have baseline FEV1 values 40–80% of predicted, <20% variation in screening FEV1 or FVC screening vs. injection visit, and no acute pulmonary complications in the prior 4 months.

Patients were excluded from both studies if they were currently treated with a baclofen pump, pregnant, nursing, or planning a pregnancy during the study, females of childbearing potential not using a reliable means of contraception, or had enrolled in another investigational study within the last 30 days. Patients were excluded from Study 1 if they had profound atrophy of the muscles to be injected (in the opinion of the investigator), previous botulinum toxin injection within 4 months of enrollment, or unstable antispasticity medication dosing during the past 30 days. Patients were excluded from Study 2 if they had multiple sclerosis or primary lateral sclerosis where progression of the disease might have confounded study results, more than one overnight hospitalization or two emergency room visits for respiratory-related event(s) in the last 12 months, or changes in corticosteroid medication dose/regimen in the last 2 months.

### 4.5. Data Analysis

All PFT analyses were based on the safety population, defined as doses patients received as opposed to doses to which they were randomized. For both studies, baseline was the last PFT observation on or before day 1 prior to the first dose of study medication. For all analyses, PFT results were compared to study baseline, even after multiple treatments (e.g., if patients were reinjected at 12 weeks, their PFT values at subsequent follow-up time points were compared back to the week 0 study baseline values instead of to week 12 values). PFT analyses were based on observed data.

FVC and FEV1 values at study baseline and change from baseline values at each follow-up visit were analyzed using a one-way analysis of variance with treatment as the factor, followed by pairwise comparisons between each onabotA dose group and placebo. Change from baseline data for FVC and FEV1 values were also tabulated by percentage of patients showing changes from baseline in 0.2 L increments. The numbers of patients in each treatment group with ≥12% and ≥0.2 L decreases from baseline in FVC or FEV1 were compared to placebo using Pearson’s chi-square tests, or, if 25% or more of the cells had expected counts were less than 5, using Fisher’s exact tests. Change from baseline FVC values were further stratified by baseline FVC quartile for placebo and onabotA 360 U and data were graphed for observation of trends.

Efficacy analyses were based on the intent to treat population, defined as all randomized patients. Missing data were imputed using the change rate method. Change from baseline in mean Ashworth scores were calculated for each treatment group and compared with the placebo group using the Wilcoxon rank-sum test.

Adverse events were summarized by group for all adverse events, events judged by investigators to be treatment related, and pulmonary function related adverse events, defined as adverse events recorded in the following Medical Dictionary for Regulatory Activities (MedDRA) categories: bronchial disorders (excluding neoplasms), lower respiratory tract disorders (excluding obstruction and infection), pleural disorders, pulmonary vascular disorders, respiratory disorders NEC, respiratory tract infections, thoracic disorders (excluding lung and pleura), upper respiratory tract disorders (excluding infections), blood gas and acid base analyses, physical examination procedures and organ system status, respiratory and pulmonary function diagnostic procedures. Adverse event analyses included all patients who received at least one dose of study medication and were based on dose actually received. Statistical significance was set at *p* < 0.05 for all tests.

### 4.6. Analysis Populations

As reinjection was permitted but not required in these studies, the post-treatment time course is not the same for all patients after week 12. Consequently, subanalyses were conducted to include patients reinjected at week 12 (Population 2), and patients reinjected at week 18 (Population 3; Study 2 only). For comparison, the overall safety population is referred to as the double-blind placebo controlled (DBPC) Population for each study.

## Figures and Tables

**Table 1:** Demographic and baseline characteristics.

Study:	Study 1			Study 2		
Treatment group	360 U	240 U	Placebo	360 U	240 U	Placebo
Total *N*	37	35	36	54	53	48
Mean age (yrs)	58.8	57.7	59.1	55.5	55.9	58.0
% Female	62	46	42	39	25	40
% Caucasian	84	83	58	70	66	71
Mean height (cm)	166.3	169.3	168.9	171.4	171.5	171.1
Baseline FVC (L), mean (range)	2.77 (1.52–4.47)	3.03 (1.45–6.38)	2.96 (1.29–4.91)	2.94 (1.30–4.87)	3.05 (1.38–5.02)	2.89 (1.25–4.82)
Baseline % predicted FVC, mean (range)	78 (53–139)	77 (44–127)	76 (29–151)	71 (47–105)	71 (37–105)	71 (52–108)
Baseline % predicted FVC, number (% of pts)						
<40%	0 (0%)	0 (0%)	1 (3%)	0 (0%)	1 (2%)	0 (0%)
40–60%	5 (14%)	4 (11%)	3 (8%)	10 (19%)	7 (13%)	10 (21%)
60–80%	16 (44%)	18 (51%)	20 (56%)	33 (61%)	31 (59%)	27 (56%)
>80%	15 (42%)	13 (37%)	12 (33%)	11 (20%)	14 (26%)	11 (23%)
Baseline FEV_1_, mean (range)	2.23 (1.25–3.63)	2.47 (1.29–5.18)	2.44 (1.23–3.94)	2.18 (1.03–4.34)	2.21 (0.71–3.37)	2.11 (0.88–3.66)
Baseline % predicted FEV_1_, mean (range)	79 (58–137)	79 (41–130)	80 (36–152)	66 (46–80)	65 (33–84)	65 (37–100)
Baseline % predicted FEV_1_, number (% of pts)						
<40%	0 (0%)	0 (0%)	1 (3%)	0 (0%)	1 (2%)	1 (2%)
40–60%	4 (11%)	6 (17%)	2 (6%)	17 (32%)	17 (32%)	13 (27%)
60–80%	18 (50%)	12 (34%)	15 (42%)	35 (65%)	33 (62%)	31 (65%)
>80%	14 (39%)	17 (49%)	18 (50%)	2 (4%)	2 (4%)	3 (6%)
FEV_1_/FVC ratio, mean (range)	0.81 (0.59–0.97)	0.82 (0.63–0.98)	0.83 (0.59–1.20)	0.74 (0.49–0.95)	0.73 (0.42–0.97)	0.74 (0.32–0.98)

**Table 2:** Forced vital capacity (FVC) mean change from study baseline in Study 1 (observed data, double-blind placebo controlled (DBPC) Population ^a^).

Treatment Group:		360 U	240 U	Placebo	360 U vs. Placebo Difference ^b^ 95% CI ^c^	240 U vs. Placebo Difference 95% CI
Study Baseline	*N*	37	35	36		
	FVC, L	2.772	3.032	2.958	−0.19 (−0.58, 0.21)	0.07 (−0.32, 0.47)
Week 1	*N*	37	35	35		
	Mean Δ	−0.001	−0.002	0.114	−0.12 (−0.27, 0.04)	−0.12 (−0.28, 0.04)
Week 3	*N*	35	35	31		
	Mean Δ	0.047	−0.057 *	0.110	−0.06 (−0.22, 0.09)	−0.17 (−0.32, −0.01)
Week 6	*N*	37	33	36		
	Mean Δ	0.003	0.029	0.126	−0.12 (−0.25, 0.01)	−0.10 (−0.23, 0.04)
Week 12	*N*	34	33	27		
	Mean Δ	−0.006 *	0.054	0.167	−0.17 (−0.33, −0.02)	−0.11 (−0.27, 0.04)
Week 13	*N*	33	32	25		
	Mean Δ	0.039	0.112	0.109	−0.07 (−0.24, 0.10)	−0.10 (−0.27, 0.08)
Week 15	*N*	33	30	28		
	Mean Δ	−0.025	0.051	0.090	−0.11 (−0.26, 0.03)	−0.04 (−0.19, 0.11)
Week 18	*N*	37	33	30		
	Mean Δ	−0.022	0.058	0.087	−0.11 (−0.26, 0.05)	−0.03 (−0.19, 0.13)

Notes: * *p*-value ≤ 0.05 for onabotA vs. placebo. Results for imputed data are consistent with the observed data. ^a^ DBPC Population: these patients received at least one dose of study medication in the DBPC study period. ^b^ Differences are active treatment minus placebo in least-squares means from a one-way ANOVA model at each visit with treatment as a main effect. ^c^ 95% confidence intervals (CI) for the differences (active treatment minus placebo) are from pairwise contrasts from a one-way ANOVA model at each visit with treatment as a main effect.

**Table 3:** FVC mean change from study baseline in Study 2 (observed data, DBPC Population ^a^).

Treatment Group:		360 U	240 U	Placebo	360 U vs. Placebo Difference ^b^ 95% CI ^c^	240 U vs. Placebo Difference 95% CI
Study Baseline	*N*	54	53	48		
	FVC, L	2.935	3.053	2.889	0.05 (−0.27, 0.36)	0.16 (−0.15, 0.48)
Week 1	*N*	53	49	48		
	Mean Δ	0.020	0.085	−0.001	0.02 (−0.08, 0.13)	0.09 (−0.02, 0.19)
Week 6	*N*	52	51	47		
	Mean Δ	−0.078 *	0.052	0.049	−0.13 (−0.24, −0.01)	0.00 (−0.11, 0.12)
Week 12	*N*	47	49	42		
	Mean Δ	−0.040	0.100	0.029	−0.07 (−0.20, 0.06)	0.07 (−0.06, 0.20)
Week 13	*N*	39	31	36		
	Mean Δ	−0.060 **	0.084	0.119	−0.18 (−0.31, −0.05)	−0.03 (−0.17, 0.11)
Week 18	*N*	47	41	43		
	Mean Δ	−0.128 ***	0.064	0.080	−0.21 (−0.33, −0.09)	−0.02 (−0.14, 0.11)
Week 19	*N*	9	14	6		
	Mean Δ	−0.064	0.189	0.025	−0.09 (−0.51, 0.33)	0.16 (−0.23, 0.56)
Week 24	*N*	46	43	43		
	Mean Δ	−0.082 ***	0.094	0.149	−0.23 (−0.37, −0.09)	−0.05 (−0.20, 0.09)
Week 30	*N*	50	47	42		
	Mean Δ	−0.046	0.088	0.061	−0.11 (−0.24, 0.02)	0.03 (−0.11, 0.16)

***, **, * *p*-value ≤ 0.001, 0.01, 0.05, respectively, for onabotA vs. placebo. ^a^ DBPC Population: these patients received at least one dose of study medication in the DBPC study period. ^b^ Differences are active treatment minus placebo in least-squares means from a one-way ANOVA model at each visit with treatment as a main effect. ^c^ 95% confidence intervals (CI) for the differences (active treatment minus placebo) are from pairwise contrasts from a one-way ANOVA model at each visit with treatment as a main effect.

**Table 4:** Number and percentage of patients with decreases of ≥12% and ≥200 mL in FVC or FEV_1_ for Study 1 (observed data).

Population:		DBPC Population ^a^			Population 2 ^b^		
Treatment Group:		360 U	240 U	Placebo	360 U	240 U	Placebo
Week 1	*N*	37	35	35	29	28	27
	*n* (%)	6 (16.2%)	4 (11.4%)	6 (17.1%)	5 (17.2%)	3 (10.7%)	3 (11.1%)
Week 6	*N*	35	35	31	27	28	25
	*n* (%)	5 (14.3%)	8 (22.9%)	3 (9.7%)	4 (14.8%)	6 (21.4%)	3 (12.0%)
Week 12	*N*	37	33	36	29	28	28
	*n* (%)	4 (10.8%)	6 (18.2%)	4 (11.1%)	4 (13.8%)	4 (14.3%)	2 (7.1%)
Week 13	*N*	34	33	27	29	28	25
	*n* (%)	6 (17.6%)	6 (18.2%)	4 (14.8%)	6 (20.7%)	5 (17.9%)	4 (16.0%)
Week 18	*N*	33	32	25	26	27	23
	*n* (%)	6 (18.2%)	7 (21.9%)	4 (16.0%)	4 (15.4%)	5 (18.5%)	4 (17.4%)
Week 24	*N*	33	30	28	27	24	25
	*n* (%)	5 (15.2%)	7 (23.3%)	5 (17.9%)	4 (14.8%)	5 (20.8%)	4 (16.0%)
Week 30	*N*	37	33	30	29	27	27
	*n* (%)	6 (16.2%)	8 (24.2%)	5 (16.7%)	4 (13.8%)	6 (22.2%)	4 (14.8%)

^a^ DBPC Population: these patients received at least one dose of study medication in the DBPC study period. ^b^ Population 2: these patients received their second injection of study medication 12 weeks after the first. Shaded columns indicate that “population 2” are separate from the DBPC population.

**Table 5:** Number and percentage of patients with decreases of ≥12% and ≥200 mL in FVC or FEV_1_ for Study 2 (observed data).

Population:		DBPC Population ^a^			Population 2 ^b^			Population 3 ^c^		
Treatment Group:		360 U	240 U	Placebo	360 U	240 U	Placebo	360 U	240 U	Placebo
Week 1	*N*	53	49	48	41	30	37	9	15	7
	*n* (%)	6 (11.3)	7 (14.3)	5 (10.4)	4 (9.8)	3 (10.0)	4 (10.8)	1 (11.1%)	2 (13.3%)	1 (14.3%)
Week 6	*N*	52	51	47	41	32	37	9	15	7
	*n* (%)	8 (15.4)	5 (9.8)	5 (10.6)	7 (17.1)	4 (12.5)	3 (8.1)	1 (11.1%)	1 (6.7%)	2 (28.6%)
Week 12	*N*	47	49	42	37	31	35	9	15	6
	*n* (%)	9 (19.1)	4 (8.2)	4 (9.5)	8 (21.6) *	1 (3.2)	1 (2.9)	1 (11.1%)	2 (13.3%)	3 (50.0%)
Week 13	*N*	39	31	36	39	31	36	--	--	--
	*n* (%)	9 (23.1)	3 (9.7)	3 (8.3)	9 (23.1)	3 (9.7)	3 (8.3)	--	--	--
Week 18	*N*	47	41	43	37	27	36	9	12	7
	*n* (%)	11 (23.4)	6 (14.6)	4 (9.3)	8 (21.6) *	5 (18.5)	1 (2.8)	3 (33.3%)	1 (8.3%)	3 (42.9%)
Week 19	*N*	9	14	6	--	--	--	9	14	6
	*n* (%)	2 (22.2%)	1 (7.1%)	1 (16.7%)	--	--	--	2 (22.2%)	1 (7.1%)	1 (16.7%)
Week 24	*N*	46	43	43	38	30	36	8	12	7
	*n* (%)	10 (21.7) *	6 (14.0)	3 (7.0)	9 (23.7) *	5 (16.7)	2 (5.6)	1 (12.5%)	1 (8.3%)	1 (14.3%)
Week 30	*N*	50	47	42	40	31	36	9	15	6
	*n* (%)	9 (18.0)	8 (17.0)	5 (11.9)	7 (17.5)	7 (22.6)	5 (13.9)	2 (22.2%)	1 (6.7%)	0 (0.0%)

* *p*-value ≤ 0.05 for onabotA vs. placebo. ^a^ DBPC Population: these patients received at least one dose of study medication in the DBPC study period. ^b^ Population 2: these patients received their second injection of study medication 12 weeks after the first. ^c^ Population 3: these patients received their second injection of study medication 18 weeks after the first. Shaded columns indicate that “population 2” are separate from the DBPC population.

**Table 6:** Change from baseline mean Ashworth scores for upper and/or lower limb in Study 1.

All Patients (*N* = 109)	Placebo (*n* = 36)	OnabotA 240 U (*n* = 36)	OnabotA 360 U (*n* = 37)	*p*-Values OnabotA 240 U vs. Placebo	*p*-Values OnaboA 360 U vs. Placebo
Baseline	3.0	2.9	2.7	0.497	0.123
Week 1	−0.4	−0.7 *	−0.9 *	0.007	0.001
Week 3	−0.5	−0.9 *	−0.9 *	0.003	0.013
Week 6	−0.2	−0.8 *	−0.8 *	<0.001	0.003
Week 12	0.0	−0.3	−0.3	0.061	0.064
Week 13	−0.3	−0.7 *	−0.7 *	0.017	0.029
Week 15	−0.3	−0.7 *	−0.7	0.031	0.055
Week 18	−0.1	−0.7 *	−0.6 *	0.001	0.013

* *p*-value ≤ 0.05 for onabotA vs. placebo.

**Table 7:** Change from baseline mean Ashworth scores for upper limb ^a^ in Study 2.

	Placebo (*n* = 48)	OnabotA 240 U (*n* = 52)	OnabotA 360 U (*n* = 55)	*p*-Values OnabotA 240 U vs. Placebo	*p*-Values OnabotA 360 U vs. Placebo
Baseline	2.65	2.34 *	2.44	0.035	0.151
Week 1	−0.28	−0.58 *	−0.61 *	0.005	0.011
Week 6	−0.39	−0.72 *	−0.93 *	0.010	<0.001
Week 12	−0.23	−0.50 *	−0.43	0.009	0.079
Week 18	−0.37	−0.60 *	−0.79 *	0.048	0.001
Week 24	−0.46	−0.53	−0.59	0.498	0.230
Week 30	−0.30	−0.45	−0.35	0.151	0.645

* *p*-value ≤ 0.05 for onabotA vs. placebo.^a^ Upper arm score was the aggregate of average scores across all upper limb muscles (i.e., elbow, wrist, fingers, and thumb).

**Table 8:** Overall rates of adverse events in Studies 1 and 2.

	Placebo	OnabotA 240 U	OnabotA 360 U
	Number (%) of Patients		
**Study 1**			
Any AEs	28/36 (77.8%)	22/36 (61.1%)	26/36 (72.2%)
Treatment-related AEs	5/36 (13.9%)	6/36 (16.7%)	5/36 (13.9%)
Serious AEs	3/36 (8.3%)	1/36 (2.8%)	3/36 (8.3%)
**Study 2**			
Any AEs	25/48 (52.1%)	30/52 (57.7%)	28/55 (50.9%)
Treatment-related AEs	4/48 (8.3%)	5/52 (9.6%)	7/55 (12.7%)
Serious AEs	7/48 (14.6%)	9/52 (17.3%)	6/55 (10.9%)

AE = adverse event.

**Table 9:** Pulmonary related adverse events in Study 1 in ≥3% of patients in any group.

Adverse Event	Placebo (*n* = 36)	OnabotA 240 U (*n* = 36)	OnabotA 360 U (*n* = 36)
Overall	11 (30.6%)	8 (22.9%)	16 (43.2%)
Nasopharyngitis	2 (5.6%)	3 (8.6%)	6 (16.2%)
Bronchitis	0 (0.0%)	1 (2.9%)	2 (5.4%)
Upper respiratory tract infection	2 (5.6%)	0 (0.0%)	2 (5.4%)
Oropharyngeal pain	0 (0.0%)	2 (5.7%)	1 (2.7%)

**Table 10:** Pulmonary related adverse events in Study 2 in ≥3% of patients in any group.

Adverse Event	Placebo (*n* = 48)	OnabotA 240 U (*n* = 53)	OnabotA 360 U (*n* = 54)
Overall	9 (18.8%)	14 (26.4%)	15 (27.8%)
Upper respiratory tract infection	3 (6.3%)	4 (7.5%)	6 (11.1%)
Nasal congestion	0 (0.0%)	2 (3.8%)	2 (3.7%)
Nasopharyngitis	1 (2.1%)	1 (1.9%)	2 (3.7%)
Cough	2 (4.2%)	3 (5.7%)	0 (0.0%)
Rhinitis	0 (0.0%)	2 (3.8%)	0 (0.0%)

## Appendix A

**Table A1:** Most frequently injected muscles in Study 1.

	360 U	240 U	Placebo	*p* Value
Week 0 total muscles injected (mean)	5.8	4.8	4.4	
Flexor digitorum superficialis	27 (73%)	23 (64%)	23 (64%)	0.625
Flexor carpi radialis	20 (54%)	21 (58%)	16 (44%)	0.502
Flexor carpi ulnaris	21 (57%)	22 (61%)	21 (58%)	0.969
Flexor digitorum profundus	26 (70%)	18 (50%)	15 (42%)	0.042
Biceps	14 (38%)	14 (39%)	13 (36%)	1.000
Week 12 total muscles injected (mean)	5.7	4.6	4.3	
Flexor digitorum superficialis	22 (59%)	17 (47%)	18 (50%)	0.566
Flexor digitorum profondus	20 (54%)	14 (39%)	13 (36%)	0.261
Flexor carpi ulnaris	17 (46%)	19 (53%)	15 (42%)	0.638
Flexor carpi radialis	16 (43%)	18 (50%)	10 (28%)	0.145
Gastrocnemius lateral	14 (38%)	5 (14%)	7 (19%)	0.049

**Table A2:** Most frequently injected muscles in Study 2.

	360 U	240 U	Placebo	*p* Value
Week 0 total muscles injected	392	386	345	
Flexor digitorum superficialis	51 (92.7%)	45 (86.5%)	45 (93.8%)	0.411
Flexor carpi radialis	52 (94.5%)	48 (92.3%)	40 (83.3%)	0.152
Flexor carpi ulnaris	49 (89.1%)	49 (94.2%)	40 (83.3%)	0.220
Biceps	48 (87.3%)	48 (92.3%)	41 (85.4%)	0.543
Flexor digitorum profundus	37 (67.3%)	37 (71.2%)	34 (70.8%)	0.905
Week 12 total muscles injected	361	329	329	
Flexor digitorum superficialis	48 (96.0%)	39 (84.8%)	42 (95.5%)	0.100
Biceps	46 (92.0%)	40 (87.0%)	36 (81.8%)	0.354
Flexor carpi radialis	43 (86.0%)	42 (91.3%)	37 (84.1%)	0.598
Flexor carpi ulnaris	43 (86.0%)	43 (93.5%)	35 (79.5%)	0.155
Flexor digitorum profundus	37 (74.0%)	30 (65.2%)	35 (79.5%)	0.315

**Table A3:** FVC mean change from study baseline in Studies 1 and 2 for Population 2 (both studies) and Population 3 (Study 2 only).

Population:		Population 2 ^a^						Population 3 ^b^		
Study:		Study 1			Study 2			Study 2		
Treatment Group:		360 U	240 U	Pbo	360 U	240 U	Pbo	360 U	240 U	Pbo
Study Baseline	*N*	29	28	28	41	32	37	9	15	7
	FVC, mL	2830	3032	2981	2935	3179	2955	3087	2788	2574
Week 1	*N*	29	28	27	41	30	37	9	15	7
	Mean Δ	−3	25	165	35	87	−6	−22	115	−23
Week 3	*N*	27	28	25	--	--	--	--	--	--
	Mean Δ	51	−20	130	--	--	--	--	--	--
Week 6	*N*	29	28	28	41	32	37	9	15	7
	Mean Δ	−11 **	30	177	−69	33	43	−122	72	−51
Week 12	*N*	29	28	25	37	31	35	9	15	6
	Mean Δ	−11 **	37	170	−50	66	80	−18	163 *	−275
Week 13	*N*	26	27	23	39	31	36	--	--	--
	Mean Δ	42	−5	103	−60 **	84	119	--	--	--
Week 15	*N*	27	24	25	--	--	--	--	--	--
	Mean Δ	−19	14	98	--	--	--	--	--	--
Week 18	*N*	29	27	27	37	27	36	9	12	7
	Mean Δ	−21	8	89	−132 ***	31	112	−127	110	−83
Week 19	***N***	--	--	--	--	--	--	9	14	6
	Mean Δ	--	--	--	--	--	--	−64	189	25
Week 24	*N*	--	--	--	38	30	36	8	12	7
	Mean Δ	--	--	--	−106 ***	51	186	36	138	−44
Week 30	*N*	--	--	--	40	31	36	9	15	6
	Mean Δ	--	--	--	−69 *	57	73	31	123	−10

***, **, * *p*-value ≤ 0.001, 0.01, 0.05, respectively, for onabotA vs. placebo. Results for imputed data are consistent with the observed data. ^a^ Population 2: these patients received their second injection of study medication 12 weeks after the first. ^b^ Population 3: these patients received their second injection of study medication 18 weeks after the first.

**Table A4:** FEV1 mean change from study baseline in Study 1 (observed data, DBPC Population ^a^).

Treatment Group:		360 U	240 U	Placebo	360 U vs. Placebo Difference ^b^ 95% CI ^c^	240 U vs. Placebo Difference 95% CI
Study Baseline	*N*	37	35	36		
	FEV1, L	2.233	2.469	2.437	−0.20 (−0.52, 0.11)	0.03 (−0.29, 0.35)
Week 1	*N*	37	35	35		
	Mean Δ	−0.044	−0.045	0.073	−0.12 (−0.25, 0.01)	−0.12 (−0.25, 0.01)
Week 3	*N*	35	35	31		
	Mean Δ	−0.009	−0.102 *	0.059	−0.07 (−0.22, 0.08)	−0.16 (−0.31, −0.01)
Week 6	*N*	37	33	36		
	Mean Δ	−0.038 *	−0.041 *	0.102	−0.14 (−0.26, −0.02)	−0.14 (−0.27, −0.02)
Week 12	*N*	34	33	27		
	Mean Δ	−0.029	−0.066	0.033	−0.06 (−0.20, 0.08)	−0.10 (−0.24, 0.04)
Week 13	*N*	33	32	25		
	Mean Δ	−0.035	−0.093	0.028	−0.06 (−0.25, 0.12)	−0.12 (−0.31, 0.06)
Week 15	*N*	33	30	28		
	Mean Δ	−0.042	−0.120	0.024	−0.07 (−0.22, 0.09)	−0.14 (−0.30, 0.01)
Week 18	*N*	37	33	30		
	Mean Δ	−0.059	−0.137	0.018	−0.08 (−0.23, 0.08)	−0.16 (−0.31, 0.00)

Note: * *p*-value < 0.05 for onabotA vs. placebo. Results for imputed data are consistent with the observed data. ^a^ DBPC Population: these patients received at least one dose of study medication in the DBPC study period. ^b^ Differences are active treatment minus placebo in least-squares means from a one-way ANOVA model at each visit with treatment as a main effect. ^c^ 95% confidence intervals (CI) for the differences (active treatment minus placebo) are from pairwise contrasts from a one-way ANOVA model at each visit with treatment as a main effect.

**Table A5:** FEV1 mean change from study baseline in Study 2 (observed data, DBPC Population ^a^).

Treatment Group:		360 U	240 U	Placebo	360 U vs. Placebo Difference ^b^ 95% CI ^c^	240 U vs. Placebo Difference 95% CI
Study Baseline	***N***	**54**	**53**	**48**		
	FEV1, L	2.183	2.206	2.111	0.07 (−0.18, 0.32)	0.09 (−0.16, 0.35)
Week 1	*N*	53	49	48		
	Mean Δ	0.011	0.054	0.024	−0.01 (−0.09, 0.07)	0.03 (−0.05, 0.11)
Week 6	*N*	52	51	47		
	Mean Δ	−0.006	0.023	0.054	−0.06 (−0.16, 0.04)	−0.03 (−0.13, 0.07)
Week 12	*N*	47	49	42		
	Mean Δ	−0.035	0.072	0.060	−0.09 (−0.20, 0.01)	0.01 (−0.09, 0.12)
Week 13	*N*	39	31	36		
	Mean Δ	−0.001	0.033	0.083	−0.08 (−0.20, 0.03)	−0.05 (−0.18, 0.08)
Week 18	*N*	47	41	43		
	Mean Δ	−0.055 **	0.059	0.139	−0.19 (−0.32, −0.07)	−0.08 (−0.21, 0.05)
Week 19	*N*	9	14	6		
	Mean Δ	−0.056	0.214	0.140	−0.20 (−0.57, 0.18)	0.07 (−0.28, 0.42)
Week 24	*N*	46	43	43		
	Mean Δ	0.000	0.023	0.071	−0.07 (−0.19, 0.05)	−0.05 (−0.17, 0.08)
Week 30	*N*	50	47	42		
	Mean Δ	0.007	0.035	0.048	−0.04 (−0.16, 0.08)	−0.01 (−0.14, 0.11)

Notes: ** *p*-value ≤ 0.01, for onabotA vs. placebo. Results for imputed data are consistent with the observed data. ^a^ DBPC Population: these patients received at least one dose of study medication in the DBPC study period. ^b^ Differences are active treatment minus placebo in least-squares means from a one-way ANOVA model at each visit with treatment as a main effect. ^c^ 95% confidence intervals (CI) for the differences (active treatment minus placebo) are from pairwise contrasts from a one-way ANOVA model at each visit with treatment as a main effect.

**Table A6:** Change from baseline mean Ashworth scores for elbow, wrist, fingers, and thumb in Study 2.

	Placebo	OnabotA 240 U	OnabotA 360 U	*p*-Values OnabotA 240 U vs. Placebo	*p*-Values OnabotA 360 U vs. Placebo
Elbow	(*n* = 45)	(*n* = 51)	(*n* = 51)		
Baseline	2.76	2.47	2.57	0.131	0.301
Week 1	−0.24	−0.53	−0.62	0.016	0.013
Week 6	−0.47	−0.60	−0.89	0.358	0.018
Week 12	−0.26	−0.40	−0.51	0.474	0.100
Week 18	−0.46	−0.51	−0.80	0.969	0.058
Week 24	−0.57	−0.42	−0.68	0.207	0.534
Week 30	−0.24	−0.40	−0.56	0.608	0.112
Wrist	(*n* = 47)	(*n* = 52)	(*n* = 54)		
Baseline	2.60	2.29	2.61	0.089	0.843
Week 1	−0.21	−0.60	−0.80	0.008	<0.001
Week 6	−0.35	−0.79	−1.19	0.011	<0.001
Week 12	−0.12	−0.43	−0.60	0.094	0.006
Week 18	−0.35	−0.54	−0.96	0.181	<0.001
Week 24	−0.36	−0.49	−0.86	0.455	0.010
Week 30	−0.34	−0.44	−0.49	0.602	0.476
Fingers	(*n* = 46)	(*n* = 49)	(*n* = 55)		
Baseline	2.80	2.47	2.40	0.060	0.018
Week 1	−0.48	−0.61	−0.49	0.369	0.970
Week 6	−0.44	−0.80	−0.81	0.062	0.029
Week 12	−0.34	−0.66	−0.28	0.060	0.700
Week 18	−0.41	−0.69	−0.62	0.164	0.132
Week 24	−0.48	−0.56	−0.29	0.818	0.200
Week 30	−0.36	−0.42	−0.11	0.809	0.124
Thumb	(*n* = 35)	(*n* = 36)	(*n* = 35)		
Baseline	2.37	2.00	1.94	0.102	0.096
Week 1	−0.23	−0.61	−0.43	0.071	0.547
Week 6	−0.35	−0.67	−0.63	0.213	0.285
Week 12	−0.29	−0.49	−0.30	0.249	0.897
Week 18	−0.38	−0.65	−0.72	0.162	0.149
Week 24	−0.49	−0.69	−0.42	0.639	0.568
Week 30	−0.29	−0.54	−0.10	0.246	0.559

**Table A7:** Serious adverse events in Study 1.

Placebo (Three Patients, Six Events)
Atrial fibrillation Thrombophlebitis, deep Syncope Accidental injury Bone fracture Joint dislocation
OnabotA 240 U (one patient, one event)
Asthma
OnabotA 360 U (three patients, three events)
Pneumonia Bone fracture Arthritis

**Table A8:** Serious adverse events in Study 2.

Placebo (Seven Patients, 13 Events)
Convulsion Anemia Hypotension Renal failure Decreased mobility Splenic abscess Cerebral infarction Prostate cancer Syncope Angina pectoris Chronic obstructive pulmonary disease Wound infection Hyponatremia
OnabotA 240 U (Nine Patients, 17 Events)
Bacteremia Cardiac failure congestive Chronic obstructive pulmonary disease Cellulitis Ischemic stroke Epilepsy Cognitive disorder Hypotension Decreased heart rate Pneumonia (one patient, two events) Abscess Hyperglycemia Meingocele Encephalitis Myocardial infarction Acute coronary syndrome
OnabotA 360 U (six patients, 13 events)
Appendicitis Convulsion (*n* = 2 patients, *n* = 5 events) Hypertension Chest pain Acute endocarditis Cardiac arrest Prostate cancer Gastroenteritis Dyspnea

**Table A9:** Pulmonary related adverse events in Study 1.

	Placebo (*n* = 36)	OnabotA 240 U (*n* = 36)	OnabotA 360 U (*n* = 36)
Overall	11 (30.6%)	8 (22.9%)	16 (43.2%)
Nasopharyngitis	2 (5.6%)	3 (8.6%)	6 (16.2%)
Bronchitis	0 (0.0%)	1 (2.9%)	2 (5.4%)
Upper respiratory tract infection	2 (5.6%)	0 (0.0%)	2 (5.4%)
Oropharyngeal pain	0 (0.0%)	2 (5.7%)	1 (2.7%)
Cough	1 (2.8%)	1 (2.9%)	1 (2.7%)
Pneumonia	1 (2.8%)	0 (0.0%)	1 (2.7%)
COPD	0 (0.0%)	0 (0.0%)	1 (2.7%)
Nasal congestion	0 (0.0%)	0 (0.0%)	1 (2.7%)
Pleurisy	0 (0.0%)	0 (0.0%)	1 (2.7%)
Asthma	0 (0.0%)	1 (2.9%)	0 (0.0%)
Rhinitis	0 (0.0%)	1 (2.9%)	0 (0.0%)
Influenza	1 (2.8%)	0 (0.0%)	0 (0.0%)
Laryngitis	1 (2.8%)	0 (0.0%)	0 (0.0%)
Musculoskeletal chest pain	1 (2.8%)	0 (0.0%)	0 (0.0%)
Pharyngitis	1 (2.8%)	0 (0.0%)	0 (0.0%)
Sinus congestion	1 (2.8%)	0 (0.0%)	0 (0.0%)
Sinusitis	1 (2.8%)	0 (0.0%)	0 (0.0%)
Wheezing	1 (2.8%)	0 (0.0%)	0 (0.0%)

COPD = chronic obstructive pulmonary disease.

**Table A10:** Pulmonary related adverse events in Study 2.

Adverse Event	Placebo (*n* = 48)	OnabotA 240 U (*n* = 53)	OnabotA 360 U (*n* = 54)
Overall	9 (18.8%)	14 (26.4%)	15 (27.8%)
Upper respiratory tract infection	3 (6.3%)	4 (7.5%)	6 (11.1%)
Nasal congestion	0 (0.0%)	2 (3.8%)	2 (3.7%)
Nasopharyngitis	1 (2.1%)	1 (1.9%)	2 (3.7%)
Cough	2 (4.2%)	3 (5.7%)	0 (0.0%)
COPD	1 (2.1%)	1 (1.9%)	1 (1.9%)
Rhinitis	0 (0.0%)	2 (3.8%)	0 (0.0%)
Bronchitis	1 (2.1%)	0 (0.0%)	1 (1.9%)
Vital capacity decreased	1 (2.1%)	0 (0.0%)	1 (1.9%)
Rhinorrhea	1 (2.1%)	1 (1.9%)	0 (0.0%)
Forced expiratory volume decreased	2 (4.2%)	0 (0.0%)	0 (0.0%)
Chest pain	0 (0.0%)	0 (0.0%)	1 (1.9%)
Dyspnea	0 (0.0%)	0 (0.0%)	1 (1.9%)
Asthma	0 (0.0%)	1 (1.9%)	0 (0.0%)
Oropharyngeal pain	0 (0.0%)	1 (1.9%)	0 (0.0%)
Pneumonia	0 (0.0%)	1 (1.9%)	0 (0.0%)
Productive cough	0 (0.0%)	1 (1.9%)	0 (0.0%)
Sinus congestion	0 (0.0%)	1 (1.9%)	0 (0.0%)
Epistaxis	1 (2.1%)	0 (0.0%)	0 (0.0%)
Sleep apnea syndrome	1 (2.1%)	0 (0.0%)	0 (0.0%)

COPD = chronic obstructive pulmonary disease.
